# The Conditions Attending Every Attack of Acute Rheumatism

**Published:** 1860-09

**Authors:** Albert T. Wheelock

**Affiliations:** Belfast, Maine


### SELECTIONS.

*The Conditions attending every Attack of Acute Rheumatism.* By ALBERT T. WHEELOCK, A. M., M. D., author of Boylston Prize Essay for 1838. Belfast, Maine.

Observations in this regard, continued through twenty years, have convinced me of the entire correctness of the following proposition, viz: THAT EVERY ACCESS OF ACUTE ARTICULAR RHEUMATISM IS IMMEDIATELY PRECEDED BY A SPECIAL CONDITION OF THE NERVOUS SYSTEM INDUCED BY MENTAL ANXIETY OR THE DEPRESSING PASSIONS. When the body is in this condition, a suppression of the sensible or insensible perspiration having taken place, the result is invariably acute rheumatism. This truth, though a simple one, is, to my mind, startling, and, without egotism, the most important pathological discovery in the present century. This track of investigation is so unusual—that is, the *rationale* of the production of a given disease—that it is not to be expected that it will be admitted without experience similar to mine, easily obtained, however, by inquiries of one's own or other's patients. It seems the more important, inasmuch as a definite cause discovered producing any one disease, affords a valuable stand-point, and an encouragement for similar investigations referring to other diseases.

Nothing of the kind, to my knowledge, had been given by any medical writer in English, and in 1855 opportunity was afforded me of making personal inquiries of several leading physicians in Paris, who stated the same was the case as far as concerned the French or German. My views were then written, and published in the *Gazette Hebdomadaire* for November of that year.

My materials, used in the investigation of the above-stated proposition, are observations of fifty cases of acute articular rheumatism. The patients were sufficiently intelligent, and the inquiries were no doubt unexpected, but at once understood, and their propriety and pertinency admitted. The cases given here, selected not entirely at random, but for the purpose of showing the disease in different aspects, were well marked, attended by fever at the outset, followed by extreme muscular prostration, and continuing generally several months.

Obs. I.—W. L., a clergyman, aged 30, married, was suffering much mental anxiety and depression, in consequence of opposition to him, and difficulties in his church. At this period he took a severe cold, in consequence of being unexpectedly out late at night, thinly clad. The following day there was a severe attack of acute rheumatism, attended by the usual symptoms, and followed by extreme muscular prostration; disease continuing more than six months.

Obs. II.—G. P., shoemaker, aged 19, an orphan, had by years of industry saved a few hundred dollars, which he was likely to lose in consequence of a mercantile failure. His anxiety deprived him of sleep, and, during the several days passed in this manner he became exposed to a sudden suppression of the perspiration by cold. An attack of acute rheumatism soon followed, with the usual symptoms.

Obs. III.—W. A. O., aged 40 master of vessel, unmarried, while on a voyage from Liverpool to Boston, the latter part of the winter season, was in a gale of wind fifteen days, and during the whole of that time constantly apprehending serious danger to his ship. During the first few days of this stormy and windy period he was much exposed to the weather, and of course very anxious concerning the fate of himself and the crew. At this time he experienced a severe cold, and a few days afterwards was prostrated by an attack of acute rheumatism. On arrival in port, being removed to hospital, he remained some months, and on discarge continued incapable for business several months more.

Obs. IV.—C. A., aged, 15, had an intimacy with a near relative, and she had become pregnant by him. Both were well known to a large circle of acquaintance, and in a situation rendering the consequences likely to be disclosed. While under this excitement and anxiety he became exposed to severe cold, rapidly followed by an attack of acute rheumatism; the more serious effects of which lasted several months.

Obs. V.—E. J., nurse, aged 30, unmarried, of much valuable experience in her vocation; was to attend her sister during an approaching confinement; the previous confinements in this case having been unusually critical. Learning by telegraph of a premature confinement in the case, she was extremely anxious to be conveyed there. While under this mental excitement, making unaccustomed exertions to succeed, neglecting her usual precautions, she experienced a sudden suppression of the perspiration, by cold, and suffered a most severe access of acute rheumatism.

OBS. VI.—B. A., clergyman, aged 40, married, nervous, sanguine temperament, bilious habit, of an unusual social disposition, accomplished education; happy in domestic relations; lost by sudden death an only child. The affliction produced nervous excitement, amounning to partial insanity. While attening to some customary manual labor during this mental disturbance, he became wet in a shower of rain, and neglecting usual precautions, experienced a severe cold. Acute rheumatism consecutively followed.

OBS. VII.—J. K., farmer, aged 20, married, living in a newly-settled district of land, and making his farm, and laboring every day of the week in order to have his crops sown seasonably, was much troubled in mind in consequence of thus disregarding the Sabbath, having strong conscientious scruples against it.—During this time, neglecting usual precautions, in consequence of his mental distraction. A severe cold was the immediate precursor of an attack of accute rheumatism, of five months' duration.

OBS. VIII.—Mr. C., merchant, aged 35, married, contracted gonorrhœa from a woman of the town; while suffering anxiety, in fear of the discovery of his faithlessness by his wife and friends, and attending to customary employment, by some neglect he experienced a severe cold, which was immediately succeeded by an access of accute rheumatism.

OBS. IX.—G. B. M., student in medicine, was candidate for house physician to a hospital, the result of the candidacy to be decided by examination and thesis. Desiring the situation on account of pecuniary necessities, as well as the honor it conferred, he exerted himself very laboriously in his studies for several months, and had much anxiety in regard to the result. As the time of examination approached, succeeding some active physical exertions, he experienced a sudden diminution of the vital heat by cold, which was immediately followed by a lengthened attack of accute rheumatism.

Obs. X.—C. H., jeweler, aged 26, had pursued his occupation successfully for two years in a new and flourishing city, when being obliged to vacate his tenement on account of the sale of the building, he found much difficulty in obtaining another. He spent several days unsuccessfully in inquiring about the place, for that object. He was delicate physically, made more so by his sedentary employment. While exerting himself in this manner, being much depressed and anxious, he became exposed to sudden cold; the consequence being a sudden attack of accute rheumatism, continuing several months, recurrences of which subsequently took place.

Obs. XI.—J. R., female, age 29, married, amiable and affectionate disposition, unusual sensibility, temperament nervous, had been for two or three weeks making preparations for an entertainment of numerous friends at her house; quite anxious, as might be expected, for everything to pass well; and during the evening of the entertainment was exposed to a strong draught of air through the opened doors, while receiving her guests. She attended to the duties of hostess, for the evening; the following day, commenced an attack of rheumatism, continuing four weeks.

Obs. XII.—B. W., a young professional man, aged 24, had an urgent engagement several miles from his residence, and was unjustly disappointed of his expected means of conveyance thither. While very much vexed, and anxious about the consequences of the delay, he went hurriedly to seek another conveyance; and obtaining a carriage, he jumped into it in a state of free perspiration, and drove rapidly off. Next day was the commencement of a severe and lengthened rheumatic fever.

Observations similar to the preceding, made during a series of years, afford no exception to the proposition above enunciated, its converse being equally found true, that no case of accute articular rheumatism has been observed not traceable to excessive mental emotions immediately previous, have settled indubitably, in my opinion, that mental emotions and cold produce with philosophical regularity and mathematical certainty the disease acute rheumatism. The connection between these phenomena is thoroughly proved.

What, then, is an important additional indication in the treatment? It is, of course, to bring into operation the requisite moral influences. The patient is to be made to understand the true nature of the disease and its cause. Though it cannot be expected that every individual shall exercise the foree necessary to the forgetting or ignoring mental agitations in these cases, yet it may be presumable that a knowledge of the real producing cause may not only prevent a recurrence of it, but will greatly assist in fortifying the sufferer against its protracted continuance. In my own experience, I have found, when patients are informed that it has been brought on themselves by a mental agitation that might seem to have been avoided or was inexcusable and needless, the disease has been shortened in its course or immediately stopped; and where there has been successive attacks, the patients had thus been apparently spared these recurrences.—*American Medical Monthly.*

